# Art Therapy for Paediatric Pain: A Scoping Review

**DOI:** 10.3390/children11060619

**Published:** 2024-05-22

**Authors:** Sofia Olaizola, Chitra Lalloo, Victoria Vickers, Lauren Kelenc, Sakib Tariq, Stephen C. Brown, Jennifer N. Stinson

**Affiliations:** 1 Child Health Evaluative Sciences, SickKids Research Institute, The Hospital for Sick Children, 686 Bay St., Toronto, ON M5G0A4, Canada; sofia.olaizola@sickkids.ca (S.O.); chitra.lalloo@sickkids.ca (C.L.);; 2 Toronto Art Therapy Institute, 8 Prince Arthur Ave., M5R 1A9, Toronto, ON M5R 1A9, Canada; 3 St. George’s University of London, Cranmer Terrace, London SW17 0RE, UK; 4 Department of Anesthesia and Pain Medicine, The Hospital for Sick Children, 170 Elizabeth St., Toronto, ON M5G 1E8, Canada; stephen.brown@sickkids.ca; 5 Anesthesiology and Pain Medicine, University of Toronto, 123 Edward St., Toronto, ON M5G 1E2, Canada; 6 Lawrence S. Bloomberg Faculty of Nursing, University of Toronto, 155 College St. Rapid Prototype Centre, Toronto, ON M5T 1P8, Canada

**Keywords:** art therapy, children, adolescents, pain, scoping review

## Abstract

Pain is common in paediatric populations and is best treated with a multi-disciplinary approach. Art therapy interventions are gaining popularity in paediatrics; however, there is limited evidence on its impact on pain outcomes in children and adolescents. The objective of this scoping review is to map current research on art therapy’s impact as an intervention in paediatric populations experiencing any type of pain (i.e., acute, recurrent, and chronic). Electronic searches were conducted by a medical librarian to identify studies that used art therapy interventions in paediatric populations with pain as an outcome measure. Four reviewers independently screened and selected articles for extraction using Covidence and data were extracted from articles using study objectives. There were five studies that met the inclusion criteria. Four of the five studies reported on pain intensity and all studies reported on emotional functioning. Findings suggest art therapy interventions can be helpful for reducing pain, anxiety, stress, and fear associated with treatment. Further, there is emerging evidence that art therapy can support the management of acute and procedural pain in children. Future research should examine the impacts of integrating art therapy interventions into the multidisciplinary management of paediatric pain.

## 1. Introduction

Pain affects children and adolescents worldwide and can become chronic if left untreated, impacting all aspects of life. Specifically, pain is estimated to affect 40% of children and adolescents globally, with 5–10% reporting that it highly interferes with daily functioning [1,2]. Pain manifests itself in various forms ranging from acute, which includes procedural pain (i.e., needle pokes, vaccinations, etc.), and chronic pain. Acute pain is defined as a short-term sudden and intense pain located to a specific area of the body and can arise from injury, or post-operatively. Procedural pain is a type of acute pain arising from medical procedures like needle pokes, injections, or other medical interventions. Acute pain types are typically treated with non-pharmacological strategies such as distraction and physical interventions. Chronic pain is defined as persistent or recurring pain that occurs beyond the healing time of any obvious pathology and occurs for more than 3 months. Causes of the high rate of chronic pain in children are multifactorial and have been shown to be influenced by gender, socioeconomic status, age, family, anxiety, and depression [2,3,4]. As such, paediatric chronic pain is multifactorial and multidimensional which necessitates a multidisciplinary approach to treatment. The recommended approach to managing both paediatric acute and chronic pain is to incorporate pharmacological, physical, and psychological strategies (i.e., a 3-P approach).

**Figure 1:**
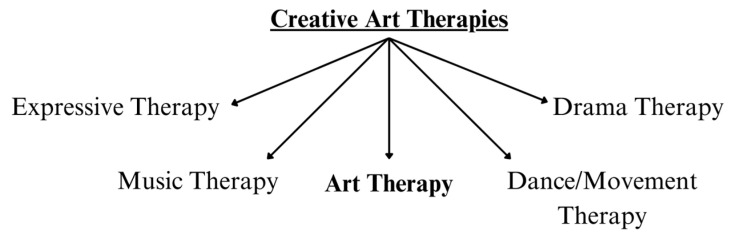
Contextualizing art therapy within the larger field of creative art therapies for medical use.

Creative art therapies are an example of a non-pharmacological approach to pain management which could include both physical and psychological strategies (see Figure 1). Within the literature, creative art therapy interventions are often used in combination (e.g., dance and music together). “Art therapy” is a specific modality under the umbrella of creative art therapies, which has gained a lot of popularity among paediatric populations [5,6]. Art therapy incorporates psychotherapy and the creative process, under the guidance of a licensed art therapist who aims to assist in the creation of art, understanding, and self-exploration [7]. In this process, thoughts and feelings that may be challenging to articulate can be expressed using imagery and colour [7]. It centers on the therapeutic relationship between an art therapist and a client with the aim of improving the client’s physical, emotional, and mental well-being [7]. The present paper focuses specifically on art therapy as a modality for paediatric pain management since it can be easily integrated into clinical care pathways complementary to existing multidisciplinary treatment plans.

Art therapy used in medical settings is focused on providing emotional support through the patient’s medical experiences and coping with physical pain and other psychosocial symptoms like anxiety and fear [8]. It goes beyond the use of visual art activities (e.g., drawing, colouring books) as a simple distraction technique during painful procedures. An example of an art therapy intervention in a clinical setting is having a patient create a self-portrait to allow themselves, the art therapist, and their medical team to better understand how their self-image has been impacted through their medical experiences.

There is evidence that distraction can be an effective technique for reducing acute paediatric pain. A meta-analysis which investigated the effectiveness of using digital technologies as a distraction for children undergoing painful procedures found clinically significant reductions in self-reported and observer-reported pain [9]. Additionally, one review looking at the effectiveness of psychological therapies (i.e., cognitive behavioural therapy) in paediatric chronic and recurring pain populations found reductions in pain intensity and frequency post-intervention [10]. Art therapy uses components from both distraction and psychological therapy techniques. Although it has been shown that distraction and psychological therapies can effectively reduce pain outcomes in paediatric populations, little is known about art therapy’s impact on paediatric pain outcomes.

There is some beginning evidence for positive effects of art therapy in adult chronic pain populations. One clinical case study with 12 patients demonstrated that by administering an art therapy intervention, patients experienced an increase in the acceptance of their chronic pain, were able to adopt a more positive outlook on their situation, and improve their communication related to their pain [11]. By engaging in art therapy, these patients were able to steer focus away from the intensity of their pain and learn to embody a meditative state of positivity to improve their health and well-being [11]. Another study described an intervention for a community art therapy group in adult chronic pain populations [12]. In this study, the intervention included twelve weekly group art therapy sessions in which members spent a portion of the session freely creating art under the guidance of an art therapist and the remaining portion in group discussion with other members [12]. More recently, a study in adults looked at the effects of a bedside art therapy intervention in patients admitted for acute care [13]. Findings from this study showed a significant reduction in pain and anxiety, and significant changes in mood [13].

Studies have also shown that art therapy can help improve the quality of life in cancer, burn, and palliative paediatric populations [14,15,16,17,18,19,20,21,22,23,24]. In paediatric oncology populations, it has been demonstrated that through interventional drawing activities, patients improved their ability to cope with adverse side effects from their cancer treatment and communicated better with medical staff and family members [23]. Similarly, by implementing an art therapy intervention in a paediatric burn population, there was an improvement in patients’ ability to cope and a decrease in negative psychosocial symptoms (i.e., anxiety) [24]. A similar review was published in paediatric populations with varying medical health conditions looking at the effectiveness of art therapy interventions and found significant increases in self-efficacy, reductions in stress, increases in coping ability, and one paper included in the review revealed reductions in acute procedural pain intensities [25,26]. Although art therapy interventions have been implemented in certain paediatric populations, evidence for its impact on children and adolescents with acute, recurrent, and/or chronic pain remains limited.

While there have been reviews investigating art therapy in paediatric cancer, burn, palliative, and other hospitalized populations and in adult pain populations, to our knowledge, none to date have focused specifically on paediatric pain as the symptom or disease. As such, a scoping review was conducted to map current research on the impact of art therapy as an intervention for paediatric populations experiencing pain (i.e., acute, chronic, recurrent). It is hoped that this review will identify gaps in knowledge on the effect of art therapy on pediatric pain management and guide future studies in the field.

## 2. Materials and Methods

The Preferred Reporting Items for Systematic Reviews and Meta-Analyses Extension for Scoping Reviews (PRISMA-ScR) was followed for our review [27]. This review was registered on the Open Science Framework; Registration DOI: https://doi.org/10.17605/OSF.IO/CY62R (accessed on 28 November 2023) [28].

### 2.1. Inclusion Criteria

To be included in this review, papers needed to report on pain outcomes related to an art therapy intervention. Importantly, the focus of the present review was art therapy alone, not all creative arts interventions including dance, music, etc. Studies were included if they met the following criteria: published in the English language, involved human participants, primary qualitative/quantitative studies, included children or adolescents (aged 0–20 years), included any types of pain (i.e., chronic, acute, recurrent), and interventional studies that implemented an art therapy intervention. Reviews (i.e., scoping review, systematic review) were included if pain was observed as an outcome measure.

### 2.2. Search Strategy & Study Selection

Our search strategy was developed by a professional medical librarian trained in knowledge synthesis. Electronic searches were performed in the following databases: Ovid MEDLINE (R) and Epub Ahead of Print, In-Process, In-Data-Review & Other Non-Indexed Citations and Daily <1946 to 11 September 2023>; Ovid Embase+Embase Classic (1974–2023 week 36); Ovid APA PsycINFO (September week 1 2023); Web of Science Core Collection All Databases (5 September 2023) via Clarivate; and Scopus (5 September 2023) via Elsevier. The search strategy consisted of both controlled vocabulary, such as the National Library of Medicine’s MeSH (Medical Subject Headings) and Emtree Subject Headings (Embase), and APA PsycINFO subject headings and keywords. No date limits were applied. A sample of our search strategy is in Appendix A.

Once all references were retrieved by the hospital librarian, a member of the research team uploaded the data to Covidence, a web-based collaboration software that streamlines production of systematic and scoping reviews, for manual screening, removing all duplicate articles [29]. A PRISMA flowchart diagram of the search and screening process will be provided to demonstrate the articles that were selected for data extraction and those that were deemed ineligible, including the reasons for ineligibility. Using Covidence, all articles were screened one-by-one using our inclusion and exclusion criteria divided amongst three team members (SO, LK, ST), with a separate reviewer responsible for assessing conflicts (JS). Each article had to be approved by two screeners. First, abstracts and titles alone were screened, then full texts, and finally those passing the full text stage were pulled for data extraction.

### 2.3. Data Extraction

The data extraction form was jointly developed by the research team, including two reviewers, to determine relevant variables. Extracted data included: author (year), country of origin, the article’s main objective, study design, population (sex and age), type of pain experienced (i.e., acute, chronic, procedural), sample size, setting (i.e., where the intervention took place), intervention administrator (i.e., certified art therapist, other, not specified), description of art therapy intervention (type, dose, duration, and frequency), goals of therapy, clinical outcomes, main results, and key recommendations.

## 3. Results

### 3.1. General Overview

**Figure 2:**
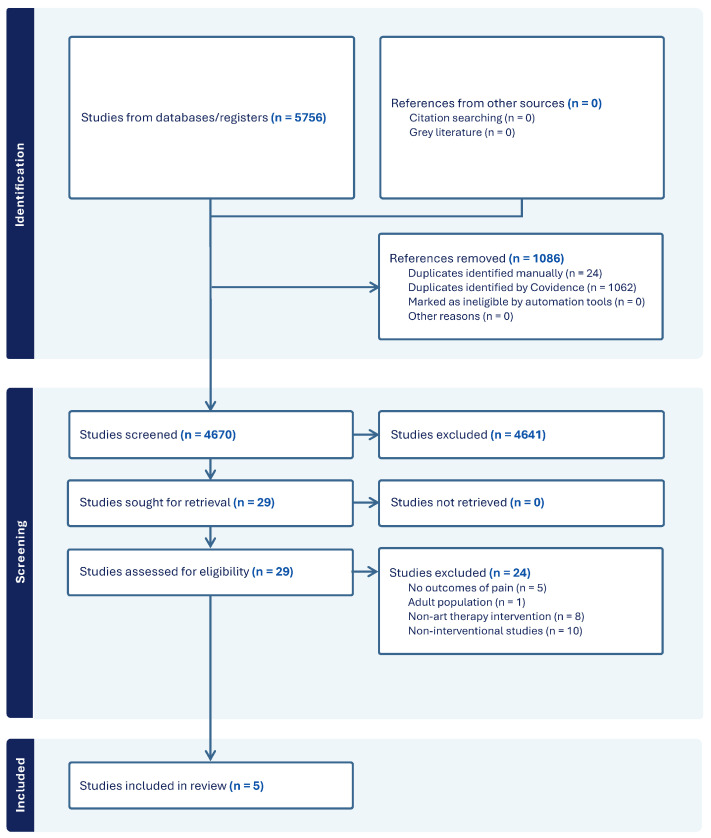
PRISMA flowchart of the screening process.

The search strategy yielded 5756 studies (see Figure 2. PRISMA flowchart of the screening process). Using Covidence, 1062 duplicates were removed, and an additional 24 duplicates were identified manually, resulting in 4670 studies to be screened. After screening titles and abstracts alone, 29 were selected for full-text screening. Of the 29 full-text studies, 24 were excluded due to being non-interventional (10), art-based activities not meeting the definition of art therapy (8), non-pain related outcomes (5), and adult population (1). Five studies were included in the final analysis, and the results are reported below. 

### 3.2. Study Participants and Design

An overview of all study characteristics can be found in Table 1. Most studies were conducted in the United States (*n* = 4; 80%) [18,26,30,31], with the remaining study conducted in India (*n* = 1; 20%) [32]. Most studies recruited participants from hospital and clinical settings (*n* = 4; 80%) [18,26,31,32], with one from a high school (*n* = 1; 20%) [30]. Patient populations included those undergoing acute procedural pain procedures (e.g., needle pokes, venipuncture) (*n* = 1; 20%) [26], those experiencing post-operative pain (*n* = 1; 20%) [32], acute pain in oncology populations (*n* = 1; 20%) [18], recurring pain (headaches) (*n* = 1; 20%) [30], and unspecified hospital populations (*n* = 1; 20%) [31]. Study designs included mixed methods (*n* = 2, 40%) [18,30], randomized controlled trials (*n* = 2, 40%) [26,32], and pre–post comparison (*n* = 1, 20%) [31]. Sample sizes varied widely across studies, from 8 to 120 participants, and participant ages ranged from 2 to 20 years old. Participant sex varied across studies, with fewer females (*n* = 2, 40%) [18,32], equal number of females and males (*n* = 1, 20%) [26], females only (*n* = 1, 20%) [30], and unspecified sex data (*n* = 1, 20%) [31].

### 3.3. Study Intervention Effectiveness on Pain Outcomes

The effectiveness of study interventions on pain outcomes can be found in Table 2. Of the visual art interventions from the five studies, three were administered by an art therapist (*n* = 3, 60%) [18,30,31], and the remaining two studies did not report intervention administrators (*n* = 2, 40%) [26,32]. Some studies did not specify the specific art therapy interventions implemented but noted that art activities included drawing and sculpting art therapies (*n* = 2, 40%) [18,31]. Other specific interventions included mindfulness interventions with individual and group drawing therapies (*n* = 1, 20%) [30], dot drawing therapy (*n* = 1, 20%) [32], and mandala-making (*n* = 1, 20%) [26]. Dose of intervention ranged from six 50 min sessions (*n* = 2) [18,30] to one-time sessions (*n* = 2) [26,32], and one study did not report how many sessions took place (*n* = 1) [31].

Findings from the studies suggest that art therapy interventions can be helpful for reducing pain, anxiety, stress, and fear associated with treatment. Most studies presented statistically significant findings in self-reported and parent-reported pain intensity over the course of the art therapy intervention (*n* = 3, 60%). Specifically, one study reported statistically significant differences in parent-reports of children’s hurt and nausea [18]. Another study showed statistically significant differences in pre- and post-session pain scores in which patients’ self-reported pain decreased [31]. Additionally, levels of both self-reported pain and anxiety decreased from pre- to post-test scores after art therapy interventions, with significant differences found [32]. One study demonstrated statistically significant reductions in momentary stress and a non-significant reduction in the number of self-reported headaches over the course of the intervention [30]. Although the remaining study noted the same downward trend in self-reported pain scores, no statistically significant differences were found for pain intensity [26]. However, the study demonstrated a significant decrease in anxiety and stress behaviours in the treatment group over time [26].

Some key recommendations that emerged from the studies were: (a) having future studies use a comparative control group to explore causal impacts with larger sample sizes, (b) ensuring that interventionists are certified art therapists, and/or clearly describing their training, (c) clearly defining outcomes of pain, in addition to mood, anxiety and well-being, (d) including prospective rather than recalled instruments, (e) including biophysical parameters like IgA levels in saliva cortisol or vital signs, and (f) focusing on clinical meaningfulness to patients rather than statistical significance alone.

## 4. Discussion

The objective of this scoping review was to identify and review current evidence for art therapy interventions on pain management in paediatric populations. We identified five research papers as eligible. The main findings from the studies show that art therapy can be helpful in reducing pain intensity, anxiety, and stress behaviours in paediatric populations. With that said, art therapy should be offered alongside established pharmacological, psychological, and physical therapy interventions to provide paediatric pain populations with the full scope of multidisciplinary care that their conditions necessitate. 

The core outcome domains recommended for clinical trials in paediatric acute pain populations include (1) pain intensity, (2) global judgement of satisfaction with treatment, (3) symptoms and adverse events, (4) physical recovery, (5) emotional response, and (6) economic factors [33]. The studies in this review reported only on two of the six recommended core outcomes which are pain intensity and emotional response. All five included papers reported on emotional response outcomes post-intervention including fear, anxiety, mood, and stress [18,26,30,31,32]. Four of the five papers also reported on pain intensity [18,26,31,32]. However, reports from other recommended domains were missing. Future studies investigating the effects of art therapy interventions in paediatric chronic pain populations should report on recommended outcomes for chronic pain trials. These include (1) pain interference with daily living, (2) pain severity, (3) overall well-being, (4) sleep, (5) physical functioning, (6) emotional functioning, and (7) adverse events [34]. 

In four out of five studies, there was an impact of art therapy on reducing pain intensity [18,26,31,32]. Similarly, in adult chronic pain populations, art therapy has been shown to have positive effects on the reduction of pain intensity [35]. For instance, a mixed-methods pilot study in an adult chronic pain population used a drawing therapy intervention and showed significant post-intervention outcomes in pain intensity [36]. Patients used drawing activities as an opportunity to modify their perceptions of their pain and to ‘take control’ over their pain, which in turn led to better coping abilities and pain reduction [36]. A similar scoping review looked at the effect of art therapy in an adult chronic pain populations and found 14 studies that demonstrated positive outcomes in pain reduction [37]. The main findings of the adult scoping review are that through art therapy-based interventions, patients can learn to draw their focus away from their current levels of pain and express themselves in a meaningful way, improving their psychosocial and physical well-being [37].

The present review also supports previous literature that has suggested that art therapy is an effective support to oncology patients, in both paediatric and adult populations. For instance, one recently published review presented seven articles that showed art therapy’s positive effects on psycho-social symptoms and overall quality of life in adults with cancer [22]. Additionally, through our screening process, there were four papers in paediatric oncology patients that presented positive outcomes in overall well-being post-art therapy interventions, based on various paediatric quality of life scales [23,38,39,40]. Art therapy had a positive impact on anxiety, fear, communication, mood, and coping ability [23,38,39,40]. These papers were excluded since they did not explicitly list pain as an outcome measure.

One challenge of this review was to distinguish between art therapy interventions and the use of artistic activities as a distraction technique (e.g., colouring a picture during a painful procedure). Notably, during the full-text screening process, two studies were excluded because the described intervention used art-based activities but did not qualify as art therapy. There is existing literature investigating the use of art-based activities as a distraction tool to reduce pain during acute pain procedures [41,42]. However, using art as a distraction should not be conflated with art therapy interventions since it lacks the crucial components of an established therapeutic relationship and intentional, specific therapeutic goals [7]. These key components should be considered when evaluating art therapy interventions in future research.

Given the consistency in the current literature of positive outcomes in physical, psychological, and social domains, associated with art therapy interventions across paediatric and adult populations, including adult pain patients, the basis for further exploration in paediatric pain patients is evident. Specifically, future work should investigate the effects of incorporating art therapy interventions as part of existing multidisciplinary treatment plans, within clinical chronic pain programs. There is research to suggest that it is helpful for acute pain patients but its effectiveness on chronic pain remains unknown.

### 4.1. Future Directions

Future research should focus on more rigorous trials with an adequate sample size and control groups. Future studies should focus on comparing art therapy interventions alone to control groups, and art therapy interventions part of a multidisciplinary approach to control groups, and their effect on managing pain and other psychosocial symptoms (i.e., anxiety, fear, etc.). Future studies can also compare art therapy to other art-based modalities. Additionally, studies should include trained art therapists and more clearly describe the training and certifications of the art therapist or person providing the therapy. Furthermore, future research should better integrate components of cultural humility and recognize practical implications of designing group medical art therapy programs across cultural contexts and backgrounds.

Next steps within our team include exploring the impact on pain and other mental health and social well-being outcomes of a 6 week group art therapy program led by an art therapy student (VV) on children and adolescents with chronic pain at the Hospital for Sick Children in Toronto. This initiative will help assess feasibility of integrating art therapy as part of a multidisciplinary paediatric pain clinic.

### 4.2. Limitations

This review has several limitations. First, eligible studies were limited only to those published in English, therefore articles that support art therapy interventions published in other languages may be missing. Additionally, art therapy interventions differed widely across all studies (i.e., frequency, length of time, intervention type, interventionist). This diversity must be considered when comparing and evaluating robustness and consistency across the results. Finally, only five papers met the criteria to be extracted for the present review which makes it difficult to draw concrete conclusions regarding art therapy’s true impact on pain outcomes and there were limited data on recurrent and chronic pain outcomes. However, the strengths of the present review include consistency of reviewers across all stages of screening and extraction. Further, the low number of eligible papers included in this scoping review is consistent with other published reviews looking at the effects of paediatric art therapy in other populations. For instance, one review investigated psychosocial outcomes of art therapy in paediatric oncology patients and obtained only seven eligible studies after an extensive review [23]. The low number of eligible papers is a testament to the potential for future work to be done in this field.

## 5. Conclusions

The aim of this scoping review was to map current research on the impact of art therapy on pain outcomes in paediatric populations. The evidence suggests a promising opportunity for art therapy to be used as a pain reduction technique in conjunction with other treatment plans in clinical settings. Most articles were on acute pain with minimal focus on recurrent and chronic pain. Given the limited number of eligible studies for this review, there remains a clear need for a pilot intervention study to be implemented in a paediatric chronic pain population, using recommendations and guidelines from current literature, to determine true effectiveness for this population. While results are promising, more research is needed using larger samples, well-controlled clinical trials, using core outcome domains recommended for acute and chronic pain trials, and using trained medical art therapists. It would also be important to explore the cost-effectiveness of these interventions.

## Figures and Tables

**Table 1:** Demographic characteristics of included articles.

Author (Year)	Country	Setting	Type of Pain	Study Design	Study Aims	Sample Size	Age	Sex (% Female)
Björling, Stevens & Singh (2019) [30]	USA	High School	Non-injury related headaches	Mixed methods participatory design	Does an art-based mindfulness intervention reduce stress and number of headaches in adolescent girls?	8	M = 15.6 Range: 14–17	100%
Madden, J., Mowry, P., Gao, D., McGuire, P., & Foreman, N. (2010) [18]	USA	Paediatric Hospital	Acute Pain (oncology/brain tumor); *Pilot RCT:* small, randomized pilot with brain tumor patients only; *Non-randomized*: all eligible hematology/oncology patients receiving CAT.	Mixed methods pilot study using repeated measures in 3 phases: (1) Small RCT with brain tumor patients randomized to creative art therapy (CAT) or volunteer’s attention (control); (2) Descriptive study of all patients that received CAT during infusions in outpatient clinic; (3) Qualitative interviews with medical and nursing staff.	Examining effect of CAT on quality of life (QOL) of brain tumor patients and patients receiving infusions in the outpatient clinic. (1) Does CAT improve reported QOL for paediatric brain tumor patients receiving chemotherapy? (2) Does CAT in a group setting improve the mood of children receiving outpatient infusions? (3) How is CAT received by providers in a busy outpatient clinic/infusion center?	Pilot RCT: 16 Non-randomized: 3232	Pilot RCT: M = 5.3 Range = 2–13 Non-randomized: M = 8.3 Range = 3–21	Pilot RCT: 25% Non-randomized: 43.75%
Metzl, E., Morrell, M., & Field, A. (2016) [31]	USA	Paediatric Hospital	Unspecified (all hospitalized patients)	Pre–post comparison design of art and music therapy (AT, MT)	Measure the effects of music and art therapy, so that improved understanding of its impact will allow art therapists, music therapists, and administrators to provide the most beneficial expressive therapy services to paediatric patients.	Unspecified Mood in 503 sessions (397 AT; 106 MT), and/or pain in 411 sessions (279 AT; 132 MT).	Range: 4–20	Unspecified
Resmy, V., & Kumar, R.N. (2020) [32]	India	Saveetha College of Nursing	Post-operative pain	RCT with pre–post study design	(1) To assess the level of anxiety and pain before art therapy; (2) To assess the effectiveness of art therapy on anxiety and pain level in post-operative children; (3) To associate the level of anxiety and pain with selected demographic variables.	60	Intervention: Range = 7–8 Control: Range = 9–10	Intervention: 36.7% Control: 43.3%
Stinley, N.E., Norris, D.O., & Hinds, P.S. (2015) [26]	USA	Laboratory Medicine Clinic	Acute procedural pain (needle pokes)	RCT	Explore the feasibility of a fast-acting mandala intervention to reduce physical pain and psychological anxiety experienced during needle sticks.	40	Intervention: M = 12 SD = 2.9 Control: M = 12.7 SD = 2.8	50%

**Table 2:** Interventions and outcomes of included articles.

Author (Year)	Intervention Administrator	Description of Intervention	Goals of Art Therapy	Clinical Outcome Measures	Results
Björling, E. A., Stevens, C., & Singh, N. B. (2019) [30]	Art Therapist	Arts-based mindfulness intervention (6, 50 min sessions including 15 min of psychoeducation, 20 min of mindfulness art sessions and 5–10 min closing activity); At end of each session either made body map or group drawing; Qualitative data at three time points.	Reduce stress and number of headaches	Perceived Stress Scale; Momentary stress assessed on a visual analog scale; Diamond Headache Questionnaire; Group Interviews at three time points—baseline, upon completion of program, and 7 weeks post-program completion.	The number of self-reported headaches decreased over the course of the intervention (T1 M = 7.38, T2 M = 4.63, T3 M = 4.13; F = 8.65; P = 0.015)’ PSS scores remained mostly stable over course of the intervention (*p* = 0.996); Momentary reports of stress showed a significant decrease from Time 1 (M = 5.12) to Time 2 (M = 3.36) but increased again at Time 3 (M = 3.88) (F = 11.87. *p* = 0.001); Qualitatively, the teens reported a reduction in stress over the course of study.
Madden, J., Mowry, P., Gao, D., McGuire, P.,&Foreman, N. (2010) [18]	Master’s-prepared, licensed dance/movement therapist with experience in music & art therapies	6 one-hour sessions, 2 sessions of each modality of creative arts therapy in pilot RCT; Non-random group had group based 1 h sessions in the infusion room group.	Improve quality of life	The PedsQL 4.0 Cancer Module; The Faces Scale; The Emotional Reactions Checklist.	Pilot RCT: Parent-report of child’s hurt (problems with having a lot of pain; *p* = 0.03), and parent-report of child’s nausea (becoming nauseated while thinking about medical treatment; *p* = 0.0061) were significantly different compared to control group and decreased over time; Due to small sample, all the child self-reports were eliminated from analysis. Non-random sample: Showed improved mood with statistical significance on the Faces Scale (*p* < 0.01), and patients were more excited (*p* < 0.05), happier (*p* < 0.02), and less nervous (*p* < 0.02) on the Emotional Responses Checklist than control group.
Metzl, E., Morell, M., & Field, A. (2016) [31]	Art and music therapists	Unspecified	Impact on mood and/or pain: (1) Is there an overall effect from art and music therapy sessions in reducing pain? (2) Is there a difference between the effect of music therapy and art therapy in reducing pain? (3) Is there an overall effect from art and music therapy sessions in improving mood? (4) Is there a difference between the effect of music therapy and art therapy in improving mood?	Wong–Baker FACES Pain Rating Scale for assessing pain and mood	When examining pre- and post-session pain scores, there was a statistically significant difference, suggesting that pain does decrease with art therapy; Specifically, a *t*-test of pain scores revealed statistically significant mean differences between pre- and post-session pain scores (*t* = 0.64 *p* = 0.0001), implying that patients’ experienced pain was reduced using the faces pain scale; There was no difference in scores by type of therapy; it was noted that those with higher levels of pain at baseline preferred MT and those with moderate to severe pain declined to participate in either therapy; A *t*-test of overall changes in mood scores revealed statistically significant mean differences between pre- and post-scores (*t* =1.31; *p* = 0.000), suggesting that patients’ moods improved, as evidenced by a change from a mean of 1.32 to 0.74 using the faces [mood] scale; A *t*-test exploring the differences of mood scores between modalities revealed statistically significant differences between pre- and post-changes in mood when comparing AT to MR (*t* = 0.011; *p* = 0.018), thereby suggesting that patients’ reported mean mood scores improved more after AT than after MT; The effect size statistic calculated as the partial eta squared for the mean differences suggested a small effect (d = 0.011); Qualitative interviews—positive about use of CAT on mood.
Resmy, V., & Kumar, R. N. (2020) [32]	Not reported	Dot drawing therapy	Reduce pain and anxiety	Numerical pain scale Five facial anxiety scale	Level of pain and anxiety reduced in the experimental group than the control group in the post test (*p* < 0.005). Pretest mean score of anxiety among post operative children in the experimental group was 4.47 ± 0.51 and mean score in the control group was 4.40 ± 0.49. The calculated student independent ‘*t*’ test (*t* = 0.513) was statistically significant. The post-test mean score of anxiety among post operative children was 1.70 ± 0.47 and the post-test mean score was 4.07 ± 0.45. The calculated student independent ‘*t*’ test value of *t* = 20.013 was statistically significant. None of the demographic variables was associated with post op pain.
Stinley, N.E., Norris, D.O., & Hinds, P.S. (2015) [26]	Not reported	Mandala-making on iPad: mandala creation actively engages patients in repetitive movements to color in and around a blank circle template using the circle as a focus; Experimental group had iPad 5 min before, during, and after procedure versus control group that watched TV only.	To determine the feasibility (high enrollment and minimal clinical workflow impact); Secondary aim to determine impact on clinical outcomes reductions of physiological (heart rate and O_2_ level) and psychological symptoms (behaviors) and subjective ratings of pain and distress; Hypothesized that providing the non-pharmacological mind/body intervention would affect physiological measures, observed patient behavior, and patient-reported anxiety and subjective pain scores.	Masimo Radical 7 pulse oximeter (HR and Blood oxygen level); Hospital Fears Rating Scale; Wong–Baker visual analogue scale (pain intensity).	Intervention was feasible with high recruitment and retention rates and minimal interruption of workflow; No statistically significant differences in pain scores; Significantly fewer treatment group participants showed stress behaviors during the needle stick procedure compared to the control group participants, *p* = 0.03; Statistically significant decrease in anxiety in the treatment group over time, *p* = 0.04, SD = 1.66, Cohen’s d = 0.8. This difference between groups indicates a greater departure from and return to baseline heart rate in the control group, whereas the average heart rate in treatment group participants remained steady; The reduction was more pronounced for those with high baseline anxiety level.

## Data Availability

No new data were created or analyzed in this study. Data sharing is not applicable to this article.

## Appendix A. Sample Search Strategy

Ovid MEDLINE(R) and Epub Ahead of Print, In-Process, In-Data-Review & Other Non-Indexed Citations and Daily <1946 to 12 September 2023>.

**#**	**Searches**	**Results**
1	exp art therapy/or (art therap * or Collage? or Colo$ring or doodl * or scribbl * or drawing or visual art? or finger paint * or painting or photograph * or sculpt * or ((Work * or play or sculpt *) adj3 (clay or model?ing clay))).mp.	449,988
2	exp Pain/or “Pain Management”/or “headache disorders”/or * burns/	519,743
3	(headache * or headache * or migraine * or neuralg *).tw,kf.	141,236
4	* Mental Disorders/th or * Mental Health Services/or (non-psychotic adj (“mental * ill * ”or “mental disorder?” or disorder?)).mp.	52,241
5	((Burning or migratory or crushing or splitting or radiating or burn?) adj2 Pain?).mp.	5160
6	(Physical * Suffer * or ache? or aching).mp.	24,223
7	(pain or abdominal pain or allodynia or application site pain or bone pain or breakthrough pain or breast tenderness or burning sensation or chronic pain or cystalgia or dysmenorrhea or dyspareunia or dysuria or eye pain or eyelid pain or female genital pain or flank pain or genital pain or gingiva pain or (headache adj2 facial pain) or hyperalgesia or hypoalgesia or inflammatory pain or inguinal pain or injection pain or injection site pain or intractable pain or jaw pain or larynx pain or limb pain or lymph node pain or mastalgia or mouth pain or musculoskeletal chest pain or musculoskeletal pain or myalgia or neuralgia or nociceptive pain or noncardiac chest pain or odynophagia or oropharynx pain or painful breathing or painful defecation or painful erection or pelvic girdle pain or pelvic pain or perineal pain or phantom pain or postoperative pain or posttraumatic pain or precordial pain or procedural pain or psychogenic pain or referred pain or retrosternal pain or salivary gland pain or scrotal pain or sinus pain or skin pain or sore throat or spinal pain or substernal pain or thorax pain or tooth pain or urethral pain or vagina pain or vein pain or visceral pain or vulvodynia).tw,kf.	817,679
8	(p$ediatric * adj3 (cancer * or neoplas * or oncology)).tw,kf.	15,371
9	or/2–8	1,184,559
10	exp adolescent/or exp child/or exp infant/or * paediatrics/or (adolescen * or babies or baby or boy? or boyhood or girlhood or child * or girl? or infan * or juvenil * or kid? or minor or minors * or neonat * or neo-nat * or newborn * or new-born * or paediatric * or paediatric * or perinat * or preschool * or puber * or pubescen * or school * or teen * or toddler? or underage? or under-age? or youth * or preteen * or user? or patient?).ti,ab,kf. or (paediatric * or paediatric * or infan * or child * or adolescen * or young).jn,jw. or (paediatric * or paediatric * or infan * or child * or adolescen * or young).in.	12,378,330
11	exp adult/or adult *.mp.	8,713,512
12	10 not 11	6,781,308
13	1 and 9 and 12	3016
14	limit 13 to (english language and humans)	1775
15	remove duplicates from 14	1772
